# Effects of a Novel Prebiotic and Postbiotic Dietary Supplement on Gut Microbiota, Intestinal Barrier Markers, and Inflammation in Healthy Dogs

**DOI:** 10.3390/vetsci13050417

**Published:** 2026-04-24

**Authors:** Chuandi Yi, Céline S. Nicolas, Ziqi Sun, Qin Wang, Tianyu Dong, Yi Wu

**Affiliations:** 1 State Key Laboratory of Animal Nutrition and Feeding, College of Animal Science and Technology, China Agricultural University, Beijing 100193, China; yichuandi2025@cau.edu.cn (C.Y.); s20253040888@cau.edu.cn (Z.S.); wangqin2025@cau.edu.cn (Q.W.); dongtianyu@cau.edu.cn (T.D.); 2 Market Unit Petfood Petcare, Virbac SA, 06510 Carros, France; celine.nicolas@virbac.com

**Keywords:** floragest, dietary transition, microbiome, gut barrier calprotectin, metagenomics

## Abstract

Although prebiotics and postbiotics support gastrointestinal health, evidence for their combined effects in dogs remains limited. This study evaluated a novel prebiotic and postbiotic supplement in healthy dogs undergoing a dietary transition. Thirty-six healthy adult dogs were randomly assigned to control group (CON, high-protein basal diet with placebo chew) or treatment group (TRT, the same basal diet with chew containing prebiotics [baobab fruit pulp and acacia gum] and postbiotics [inactivated *Lactobacillus acidophilus* and selected yeast fractions]) for a 28-day formal trial following a 7-day adaptation period. The primary outcomes evaluated included clinical fecal scores, specific biomarkers of intestinal barrier function and inflammation, fecal short-chain fatty acids, and microbiota structure. Following the 7-day adaptation, formal trial baseline, fecal scores were already within the healthy range and remained optimal without differing between groups throughout the study. Compared with CON, the TRT group showed lower fecal calprotectin and serum diamine oxidase levels, and higher fecal butyrate (*p* < 0.05). Metagenomic analysis revealed increased abundances of Bacteroidota, Oscillospiraceae, Prevotellaceae, and *Prevotella* in TRT (*p* < 0.05). Overall, in healthy dogs, this supplementation was associated with favorable microbiota modulation and modulated biomarkers of intestinal barrier and inflammation within normal ranges, without altering clinical fecal endpoints.

## Simple Summary

Diet is important for keeping a dog’s digestive system healthy. Prebiotics and postbiotics are helpful ingredients often used in pet food. In this study, we tested a new supplement containing these ingredients in healthy dogs switching to a new diet. Because the dogs were already healthy, their bowel movements remained normal throughout the study. However, laboratory tests suggested that the supplemented dogs may experience positive changes in specific blood and stool tests related to gut inflammation and barrier function, alongside a potential increase in beneficial gut bacteria. These findings suggest that this combination might help maintain a healthy gut environment in healthy dogs, though further studies are needed to confirm its benefits in dogs with digestive issues.

## 1. Introduction

Canine gastrointestinal health is a cornerstone of overall well-being in dogs, influencing not only nutrient absorption and digestion but also immune function, metabolic regulation, and neurological processes. The intestinal tract harbors a dynamic ecosystem of microbiota, which plays a pivotal role in maintaining intestinal homeostasis by synthesizing essential metabolites and defending against pathogens [1]. Dysbiosis, characterized by an imbalance in gut microbial composition, has been linked to a spectrum of disorders in dogs, including chronic enteropathies, obesity, immune-mediated diseases, and even behavioral abnormalities [2]. Furthermore, a dog’s microbial exposure and subsequent gut health are closely linked to environmental and feeding-associated factors. For instance, feeding practices and hygiene conditions, such as pet bowl management, have been shown to significantly influence microbial contamination and modulate the microbial ecosystems surrounding domestic dogs [3]. Given the continuous external microbial exposure influenced by diet type and domestic management, strategies to optimize canine gut health internally have garnered significant attention in veterinary science and pet nutrition research.

Diet is widely recognized as one of the most fundamental and effective factors in driving the composition and metabolic activity of the canine gut microbiome [4]. Specifically, varying dietary fiber sources have been demonstrated to significantly modulate fecal microbiota composition and enhance short-chain fatty acid (SCFA) production in dogs, providing a strong biological plausibility for fiber-based nutritional strategies [5]. Building upon this conceptual framework, recent therapeutic interventions focus on modulating the gut microbiome through probiotics, postbiotics, and prebiotics. Probiotics, defined as live microorganisms (e.g., *Lactobacillus* and *Bifidobacterium* strains), can competitively inhibit pathogens, enhance epithelial barrier function, and stimulate immunoglobulin production [6]. Postbiotics, non-viable microbial cells or metabolites (e.g., inactivated bacteria, cell wall fragments, exopolysaccharides), offer advantages in stability and safety, exerting immunomodulatory and anti-inflammatory effects without colonization requirements [7]. Prebiotics, mostly fermentable dietary fibers, serve as substrates for beneficial bacteria, promote short-chain fatty acid (SCFA) synthesis, and contribute to a balanced gut flora [8]. A newly developed supplement incorporated both prebiotics (baobab fruit pulp and acacia gum) and postbiotic components (inactivated *L. acidophilus* and specific yeast fractions). The prebiotics synergistically supported microbial balance and gastrointestinal function in vitro [9]. Additionally, a study demonstrated that dietary supplementation with the selected postbiotics (inactivated *L. acidophilus*) enhanced gut barrier integrity and innate immune responses in broiler chickens [10]. Another study in weaned piglets reported that supplementation with the combination of selected yeast strain fractions (from *Saccharomyces cerevisiae* and *Cyberlindnera jadinii*) improved gut microbial resilience and growth performance following *E. coli* challenge [11]. Furthermore, this mix of selected prebiotics and postbiotics has demonstrated efficacy in the simulator of the canine intestinal microbial ecosystem through improving SCFA production and increasing the abundance of beneficial bacteria [9].

However, while these in vitro and non-canine in vivo models provide a promising mechanistic foundation, their direct translational value to healthy adult dogs remains to be explicitly demonstrated. Most previous studies predominantly examined single categories of bioactives or relied on laboratory models, leaving a gap in our understanding of combinatorial effects in live canine subjects. Unlike conventional fiber-based interventions that typically provide generic fermentable substrates, this specific formulation integrates complex polysaccharides with potentially immunomodulatory postbiotic fractions to concurrently support microbial metabolic capacity and mucosal barrier resilience. Therefore, the present study aims to evaluate selected fecal, serum, and microbiota-associated indicators of gut health in healthy adult dogs following supplementation with this novel prebiotic–postbiotic blend during a dietary transition and short-term feeding period.

## 2. Materials and Methods

### 2.1. Animals

All animal procedures in this study were approved by the Institutional Animal Care and Use Committee of China Agricultural University (No. AW82114202-1-1). A total of 36 healthy dogs were included in this study. All animals were over 1 year old and had normal body condition scores. Further details like breed and age are provided in Table S1. None of the animals had a history of immune-mediated diseases, allergies, or other conditions that could lead to chronic gastrointestinal dysfunction, including liver disease, pancreatic insufficiency, metabolic disorders, parasitic infections, or kidney diseases. None of these dogs had been administered medications, dietary interventions, antibiotics, or immunosuppressive drugs, nor had they undergone surgery within three months prior to the study. Dogs in pregnancy or lactation, or those unable to eat or be fed orally, were excluded from the experiment.

During the experiment, each dog was raised separately in a 1.2 × 1.5 m^2^ animal house, with a 1.2 × 3.0 m^2^ activity field outside each animal house. A dedicated caretaker conducted 2 h of supervised outdoor activity with the dogs daily. The animal rooms were kept ventilated and clean through daily cleaning and routine disinfection in accordance with standard sanitation protocols. During the study, dogs were allowed free access to water. Regarding feeding management, a daily allowance of the basal diet was provided based on body size to meet maintenance requirements (500–600 g/day for large dogs and 200–300 g/day for small dogs). To minimize human interference and reduce handling stress under standard husbandry protocols, daily food refusals were not quantitatively recorded. Therefore, the exact individual daily feed intake could not be calculated. However, overall energy balance and nutritional adequacy were indirectly evaluated by monitoring body weight on day 0 and day 28. Dogs were closely monitored for behavior, appetite, and fecal condition.

### 2.2. Study Design

A total of 36 healthy dogs were randomly assigned to either a control group (CON) or a treatment group (TRT), with 18 dogs per group. This sample size was determined a priori based on precedents from published canine dietary and microbiome intervention studies [12]. Randomization was performed using a computer-generated random number sequence, stratified by body weight (≥15 kg and <15 kg) and sex to ensure a balanced distribution. To ensure allocation concealment, the group assignments were coded and maintained by an independent coordinator who was not involved in daily animal care or data collection. Each group initially comprised 9 dogs weighing ≥15 kg (5 males and 4 females) and 9 dogs weighing <15 kg (4 males and 5 females). However, during the data analysis phase on the bioinformatics platform, the day 0 metagenomic model construction for one dog in the CON group failed due to a programmatic error. To maintain strictly balanced group sizes and reduce potential dispersion bias in distance-based multivariate analyses, the day 0 data of one additional dog in the TRT group, closely matched for body weight and physiological condition, was randomly excluded. Preliminary evaluation showed that the overall microbial community structure and the absence of baseline differences between groups were unchanged regardless of whether this sample was included or excluded. The day 28 metagenomic data and all clinical datasets for both groups were not affected by this exclusion. From the beginning of the study, all dogs switched to a high-protein basal diet and remained with this diet throughout the entire study (Table 1). The study consisted of an initial 7-day dietary adaptation phase followed by a 28-day primary evaluation phase. During the study, dogs in the CON group received a placebo soft chew daily, while those in the TRT group were orally administered the prebiotic and postbiotic supplement (soft chew) once daily. To maintain the double-blind design, the placebo soft chews were manufactured to be identical in size, shape, color, and packaging to the active supplement. To ensure a consistent relative exposure to the active ingredients across different body sizes, the supplement dose was adjusted according to the established weight stratification: dogs weighing <15 kg received a 2 g chew, whereas dogs weighing ≥15 kg received a 4 g chew. Both interventions commenced on day 1 of the pre-trial period and continued throughout the primary evaluation phase. Consequently, it is important that the “day 0” sampling point represents the baseline of the primary evaluation phase, but it was conducted strictly after the dogs had already undergone 7 days of simultaneous dietary transition and supplement or placebo exposure.

The supplement tested was provided by Virbac China (Suzhou, China). The dosage was strictly stratified by body weight: dogs weighing <15 kg received a 2 g chew, whereas dogs weighing ≥15 kg received a 4 g chew daily. The active ingredients in the supplement included yeast powder (8.5%), baobab pulp powder (5.0%), heat-killed *Lactobacillus acidophilus* (*L. acidophilus*) (1.5%), and acacia gum. The tolerance of the supplement had previously been tested by the supplier in 24 dogs, at one and five times the recommended dose for 28 days, with no side effects reported. Still, during the dietary adaptation period and throughout the study duration, the health status and behavior of the animals were observed to confirm their safety.

After this 7-day dietary adaptation period (pre-trial period), during which the fecal score of each animal was noted, blood and fecal samples were collected on day 0 and day 28 of the experiment. Fresh fecal samples (10–20 g) were collected from each dog and immediately stored in cryovials at −80 °C. Moreover, 4 mL of blood was collected from the cephalic vein of each dog into non-anticoagulant tubes. After allowing the blood to clot for 30 min, samples were centrifuged at 1500× *g* and 4 °C for 20 min, and serum in the supernatant was collected and stored at −20 °C.

### 2.3. Sample Analysis

#### 2.3.1. Body Weight and Fecal Score

On day 0 and day 28 of the experiment, the body weight of each dog was measured and recorded. During the study (day 0 to day 28) and the 7-day dietary adaptation period preceding the study (pre-trial period), trained personnel observed the fecal condition of each dog and conducted fecal scoring based on the Waltham scoring system (a nine-point ordinal scale from 1 to 5, with half-points), with scores from 2 to 3 indicating a healthy intestinal status: a score of 5 indicated watery feces with a liquid consistency; a score of 4 represented soft, unformed stools; a score of 3 corresponded to soft, formed, and moist feces that retained their shape; a score of 2 denoted firm, formed, and dry stools with structural integrity; and a score of 1 described hard, dry feces composed of small, compact masses. Fecal consistency was assessed by a single trained investigator through direct observation of freshly voided fecal samples throughout the entire study period. This approach was specifically chosen to eliminate inter-observer variability and ensure maximal intra-observer consistency. Furthermore, all laboratory personnel conducting the subsequent serum, fecal, and metagenomic analyses were completely blinded to the treatment allocation until the data analysis phase.

#### 2.3.2. Intestinal Inflammation and Permeability Indices

The intestinal barrier indicators included the serum biomarkers: lipopolysaccharide (LPS), diamine oxidase (DAO), intestinal fatty acid-binding protein (I-FABP), and zonulin. The intestinal inflammation markers included fecal calprotectin and fecal alpha-1 antitrypsin (α1-AT). All indices were measured using commercially available canine-specific ELISA kits sourced from Jiangsu Meimian Industrial Co., Ltd. (Yancheng, China), in accordance with the instructions of the manufacturer. These kits have been structurally validated by the manufacturer for use in canine biological matrices (serum and feces).

#### 2.3.3. Fecal Odor Indices

Fecal indole was analyzed following a standardized sample preparation and chromatographic procedure. Fecal samples were weighed and homogenized in centrifuge tubes, and 2 mL of dichloromethane: acetone (1:1, *v*/*v*) was added. The mixture was vortexed and subjected to ultrasonication for 30 min. Samples were then centrifuged at 7200× *g* for 5 min, and the supernatant was filtered through a 0.45 µm membrane prior to injection.

Indole quantification was performed using an Agilent 7890A gas chromatography system (Agilent Technologies, Santa Clara, CA, USA). Chromatographic conditions were as follows: column, HP-5 (30 m × 0.32 mm × 0.25 µm); initial oven temperature, 180 °C for 10 min, ramped at 30 °C/min to 250 °C and held for 2 min; injector temperature, 250 °C; carrier gas, nitrogen at 1.0 mL/min; split injection, split ratio 10:1; detector, FID at 250 °C.

#### 2.3.4. Fecal Short-Chain Fatty Acids

SCFAs were quantified using gas chromatography–mass spectrometry (GC-MS; Thermo Trace 1310–ISQ 7000, Thermo Fisher Scientific, Waltham, MA, USA). Initially, a standard solution containing SCFAs was prepared. Subsequently, 100 mg of fresh fecal sample was weighed and mixed with 450 μL of methanol and 50 μL of 2-ethylbutyric acid (1000 μg/mL) as an internal standard, followed by cryogenic sonication (Ningbo Scientz Biotechnology Co., Ltd., Ningbo, China). The samples were then centrifuged at 13,000× *g* for 15 min to collect the supernatant (Eppendorf Biotechnology International Trade Co., Ltd., Shanghai, China). The supernatant was treated with 50 mg of anhydrous sodium sulfate and further centrifuged at 13,000× *g* for 15 min at 4 °C. The resulting supernatant was collected and analyzed using the aforementioned Thermo Trace 1310–ISQ 7000 system.

#### 2.3.5. Fecal Metagenomic Analysis

DNA was extracted from dog fecal samples using the FastPure Stool DNA Isolation Kit (MJYH, Shanghai, China), and DNA integrity was verified by 1% agarose gel electrophoresis. Genomic DNA was fragmented to ~350 bp using a Covaris M220 focused-ultrasonicator(Covaris, Woburn, MA, USA), followed by paired-end library construction with the NEXTFLEX Rapid DNA-Seq kit (Bioo Scientific, Austin, TX, USA). Metagenomic sequencing was performed on the Illumina NovaSeq platform (Illumina, San Diego, CA, USA).

Raw reads were quality-filtered using fastp (v0.23.0) by trimming adapters and removing reads shorter than 50 bp or with an average quality score <20. Subsequently, host DNA contamination was removed by aligning the filtered reads to the canine reference genome (Canis lupus familiaris) using BWA (v0.7.17). Following quality control and host read removal, the clean read counts ranged from 53,305,292 to 94,546,024 reads per sample (raw reads ranged from 53,804,270 to 95,372,992 reads per sample). High-quality reads were assembled using MEGAHIT (v1.2.9), and contigs ≥300 bp were retained. Open reading frames (ORFs) were predicted using Prodigal (v2.6.3), and genes ≥100 bp were translated into amino acid sequences. Predicted genes were clustered using CD-HIT (v4.6.1) at 90% sequence identity and 90% coverage to construct a non-redundant gene set, with the longest sequence from each cluster selected as the representative. Clean reads were aligned to the non-redundant gene set using SOAPaligner (v2.21; 95% identity), and gene abundance was calculated for each sample. To account for variations in sequencing depth across samples, gene abundances were normalized to relative abundances prior to downstream taxonomic and functional analyses; no rarefaction procedures were applied. Taxonomic annotation was performed by aligning amino acid sequences against the NR database (version 20230830) using DIAMOND (v2.0.13; BLASTP, e-value ≤1 × 10^−5^), and species abundance was estimated by summing the abundances of genes assigned to each taxon.

Functional annotation of the gut microbiome was performed using the KEGG(version 20230830) and CAZy (v12) databases. For KEGG analysis, annotated genes were classified into Level 1 and Level 2 pathways, and pathway-level relative abundances were calculated for descriptive purposes. Group differences in microbial pathways were assessed at KEGG Level 3. For CAZy analysis, carbohydrate-active enzyme (CAZyme) genes were identified by homology to the CAZy database, and family-level relative abundances were calculated. CAZy families with a mean relative abundance ≥0.1% in both groups were subjected to differential analysis. The top 10 different CAZy families were visualized, and all significant families are provided in Supplementary Table S2.

### 2.4. Statistical Analysis and Visualization

Microbial sequencing data were visualized using R. Bar plots and heatmaps of bacterial community composition were generated using the ggplot2 and vegan packages, respectively. Differential bacterial species between the two groups were identified using the LEfSe analysis with an LDA score >3. Differential KEGG pathways were determined by the Wilcoxon rank-sum test with FDR correction. Other statistical analyses were performed using IBM SPSS Statistics 27.0 (Chicago, IL, USA), with each animal considered an experimental unit. Data normality was assessed using the Shapiro-Wilk test. To properly account for individual baseline variability in the two-timepoint design, continuous biological variables (including body weight, serum biomarkers, fecal odor compounds, and fecal short-chain fatty acids) measured at day 28 were evaluated between the treatment and control groups using an analysis of covariance (ANCOVA), with the corresponding day 0 baseline value included as a covariate. Non-normally distributed data and other ordinal variables were evaluated using the independent-samples Kruskal-Wallis test, followed by Dunn’s post hoc test with Bonferroni correction for all pairwise comparisons. Results are presented as mean ± SEM. Group differences were evaluated using 95% CIs and effect sizes, including partial eta-squared (partial η^2^) for ANCOVA. Statistical significance was set at *p* < 0.05, and 0.05 ≤ *p* < 0.10 was considered a trend.

## 3. Results

### 3.1. Fecal Score During the Dietary Adaptation Period (Pre-Trial Period)

Table 2 summarizes the fecal assessment data from the 7-day pre-trial phase, during which all dogs underwent a dietary adaptation to the high-protein basal diet while receiving their respective treatments. On day one, no statistical divergence was observed between the cohorts; scores across all subjects exceeded 3. Subsequently, assessments on days three (*p* = 0.03, Table 2) and four (*p* = 0.04, Table 2) revealed significantly lower scores in the TRT group compared with the CON group. By the end of the 7-day pre-trial stage, fecal consistencies had normalized and were comparable across both groups.

### 3.2. Fecal Score and Body Weight

In the CON group, the mean (±SEM) fecal score was 2.78 ± 0.14 and 2.53 ± 0.13 on day 0 and day 28, respectively. On day 0 and day 28, the fecal score of dogs in the TRT group was 2.75 ± 0.16 and 2.47 ± 0.12, respectively. The fecal score did not significantly differ between the two groups either on day 0 (*p* = 0.90, Table 3) or day 28 (*p* = 0.75, Table 3). Regarding body weight, the initial mean values on day 0 were 14.70 ± 1.22 kg for the CON group and 15.10 ± 1.33 kg for the TRT group. At the conclusion of the primary evaluation phase on day 28, the mean body weights were 15.49 ± 1.25 kg and 15.26 ± 1.37 kg for the CON and TRT groups, respectively. However, after accounting for baseline differences, the body weight of the TRT group was significantly lower than that of the CON group (*p* < 0.001, partial η^2^ = 0.470, 95% CI: [−0.947, −0.429], Table 3). Importantly, the absolute difference was small, and all subjects maintained stable body weight within a healthy physiological range without clinical weight loss.

### 3.3. Effects of the Test Supplement on Intestinal Barrier and Inflammation

**Figure 1:**
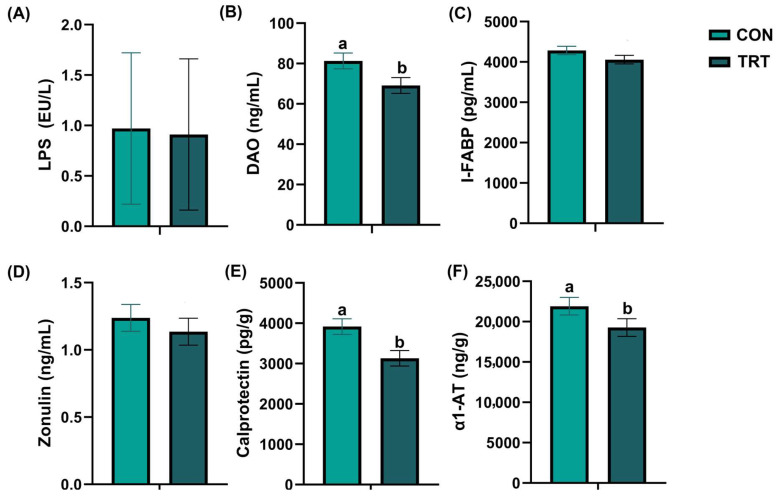
Effects of the supplement on the intestinal barrier and inflammation. (**A**) Serum LPS content. (**B**) Serum diamine oxidase (DAO) levels. (**C**) Serum intestinal fatty acid-binding protein (I-FABP) levels. (**D**) Serum zonulin levels. (**E**) Fecal calprotectin levels. (**F**) Fecal α1-antitrypsin (α1-AT) levels in the control group (CON, light green) and the treatment group (TRT, dark green). Values are expressed as mean ± SEM (CON, *n* = 18; TRT, *n* = 18). ^a,b^ Different superscript letters denote a statistically significant difference (*p* < 0.05).

The results of canine intestinal inflammation and barrier indices are shown in Figure 1. On day 28, after adjusting for initial baseline variance using ANCOVA, the serum DAO levels (*p* = 0.048, partial η^2^ = 0.113, 95% CI: [0.088, 22.596], Figure 1B) and fecal calprotectin levels (*p* = 0.007, partial η^2^ = 0.200, 95% CI: [229.747, 1342.859], Figure 1E) of dogs in the TRT group remained significantly lower than those of dogs in the CON group. Other parameters, including iFABP (*p* = 0.212, partial η^2^ = 0.047, 95% CI: [−114.619, 497.576]), LPS (*p* = 0.591, partial η^2^ = 0.009, 95% CI: [−0.274, 0.158]), zonulin (*p* = 0.478, partial η^2^ = 0.015, 95% CI: [−0.186, 0.389]), and A1AT (*p* = 0.102, partial η^2^ = 0.079, 95% CI: [−549.553, 5833.878]), did not show significant differences between the two groups at this time point.

### 3.4. Effects of the Supplement on Fecal Odor Compounds

**Figure 2:**
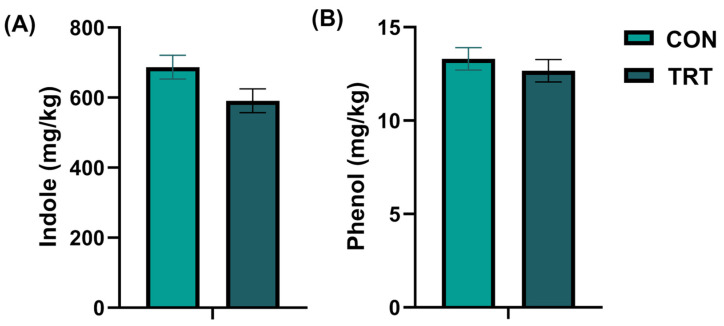
Changes in fecal odor indicators on day 28. (**A**) Fecal indole levels. (**B**) Fecal phenol levels in the control group (CON, light green) and the treatment group (TRT, dark green). Values are expressed as mean ± SEM (CON, *n* = 18; TRT, *n* = 18).

The quantification results of canine fecal odor compounds are shown in Figure 2. On day 28, after adjusting for baseline variance using ANCOVA, the indole level in the feces of the TRT group tended to be lower compared with the CON group (*p* = 0.057, partial η^2^ = 0.106, 95% CI: [−2.957, 195.554], Figure 2A), while no significant difference was observed in phenol levels (*p* = 0.481, partial η^2^ = 0.015, 95% CI: [−1.130, 2.350]).

### 3.5. Concentrations of Fecal Short-Chain Fatty Acids

**Figure 3:**
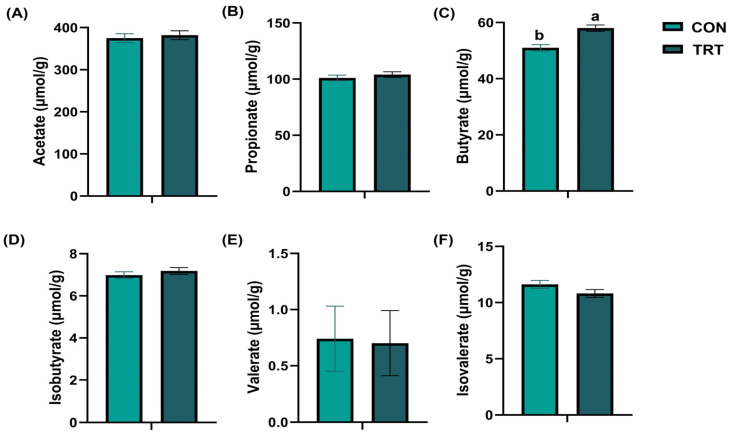
Changes in fecal short-chain fatty acids on day 28. (**A**) Fecal acetate levels (**B**) Fecal propionate levels. (**C**) Fecal butyrate levels. (**D**) Fecal isobutyrate levels. (**E**) Fecal valerate levels. (**F**) Fecal isovalerate levels in the control group (CON, light green) and the treatment group (TRT, dark green). Values are expressed as mean ± SEM (CON, *n* = 18; TRT, *n* = 18). ^a,b^ Different superscript letters denote a statistically significant difference (*p* < 0.05).

The results of canine SCFAs are shown in Figure 3. On day 28, after adjusting for initial baseline variance using ANCOVA, the butyrate level in the feces of the TRT group was significantly higher than that in the feces of the CON group (*p* = 0.001, partial η^2^ = 0.367, 95% CI: [−10.333, −3.776], Figure 3C). Levels of fecal acetate (*p* = 0.649, partial η^2^ = 0.006, 95% CI: [−37.129, 23.437]), propionate (*p* = 0.443, partial η^2^ = 0.018, 95% CI: [−10.167, 4.549]), isobutyrate (*p* = 0.389, partial η^2^ = 0.023, 95% CI: [−0.660, 0.264]), valerate (*p* = 0.378, partial η^2^ = 0.024, 95% CI: [−0.047, 0.121]), and isovalerate (*p* = 0.124, partial η^2^ = 0.070, 95% CI: [−0.232, 1.833]) in the TRT group did not significantly differ from those in the CON group.

### 3.6. Fecal Metagenomic Profiling of Gut Microbiota

#### 3.6.1. Microbial Diversity and Overall Community Structure

**Figure 4:**
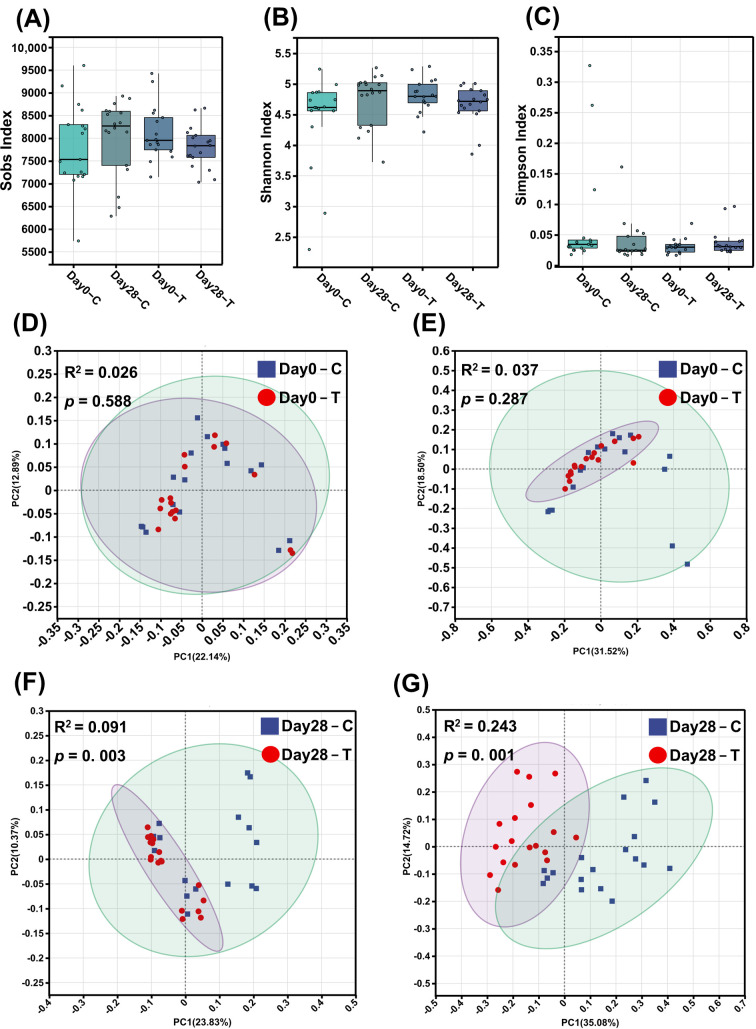
Gut microbial diversity and community structure of the control (CON, C) and treatment (TRT, T) groups on day 0 and day 28. (**A**) Sobs index. (**B**) Shannon index. (**C**) Simpson index. (**D**,**E**) Principal coordinates analysis (PCoA) of binary Jaccard and Bray–Curtis distances, respectively, on day 0. (**F**,**G**) PCoA of binary Jaccard and Bray–Curtis distances, respectively, on day 28. Bray–Curtis and binary Jaccard distances were calculated based on species-level abundance data. R^2^ and *p*-values were determined using Adonis (permutational multivariate analysis of variance). Values are expressed as mean ± SEM (day 0: CON, *n* = 17; TRT, *n* = 17; day 28: CON, *n* = 18; TRT, *n* = 18).

Fecal samples collected at days 0 and 28 underwent metagenomic sequencing. It should be noted that while all 36 dogs completed the physiological and biochemical assessments (*n* = 18 per group), one day-0 fecal sample from the CON group failed during the metagenomic library preparation process. To maintain balanced group sizes and account for weight stratification at baseline, the day-0 metagenomic data of a randomly selected, weight-matched dog from the TRT group was also excluded prior to analysis. Because no anomalies occurred during the day-28 library preparation, all 36 samples were successfully sequenced for the end-of-trial time point. Consequently, the metagenomic analysis includes 17 samples per group at day 0 and 18 samples per group at day 28. α- and β-diversity evaluations contrasting the CON and TRT groups at these intervals are depicted in Figure 4. α-diversity metrics, including Sobs, Shannon, and Simpson indices, remained statistically equivalent throughout the trial duration (Figure 4A–C). To investigate the supplement’s impact on gut microbial composition, principal coordinates analysis (PCoA) was applied to species-level data employing Bray-Curtis and binary Jaccard dissimilarity indices. At baseline (day 0), Adonis analyses indicated no substantial divergence in microbial architecture between the cohorts (binary Jaccard: *p* = 0.588; Bray-Curtis: *p* = 0.287) (Figure 4D,E). Post-supplementation assessments at day 28 demonstrated significant structural shifts in the gut microbiota between the CON and TRT groups, as evidenced by Adonis tests for both binary Jaccard (R^2^ = 0.091, *p* = 0.003) and Bray-Curtis (R^2^ = 0.243, *p* = 0.001) distances (Figure 4F,G).

#### 3.6.2. Taxonomic Composition and Differential Microbial Taxa

**Figure 5:**
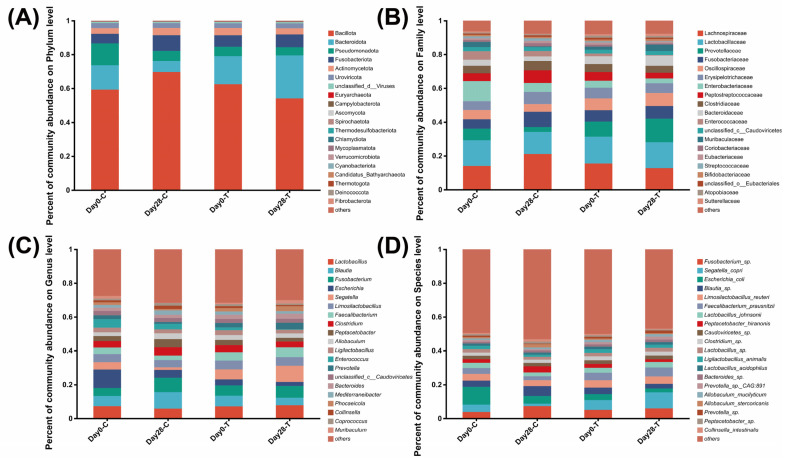
Relative abundance of bacterial taxa at the phylum (**A**), family (**B**), genus (**C**), and species (**D**) levels in the control (CON) and treatment (TRT) groups on day 0 and day 28. Values are expressed as mean ± SEM (day 0: CON, *n* = 17; TRT, *n* = 17; day 28: CON, *n* = 18; TRT, *n* = 18).

The relative abundance of fecal bacteria was measured at the phylum, family, genus, and species levels (Figure 5). Overall, the core microbial community was dominated by the phyla Bacillota, Bacteroidota, Fusobacteriota, Pseudomonadota, and Actinomycetota across all samples (Figure 5A). At the family level, Lactobacillaceae, Lachnospiraceae, Prevotellaceae, Fusobacteriaceae, and Oscillospiraceae constituted the major proportion of the microbiota (Figure 5B). The predominant genera included *Escherichia*, *Lactobacillus*, *Blautia*, *Enterococcus*, and *Limosilactobacillus* (Figure 5C). At the species level (Figure 5D), the most abundant taxa across the cohorts included *Escherichia coli*, *Segatella copri*, *Limosilactobacillus reuteri*, *Fusobacterium* sp., and *Faecalibacterium prausnitzii*.

#### 3.6.3. Identification of Differential Microbial Taxa

**Figure 6:**
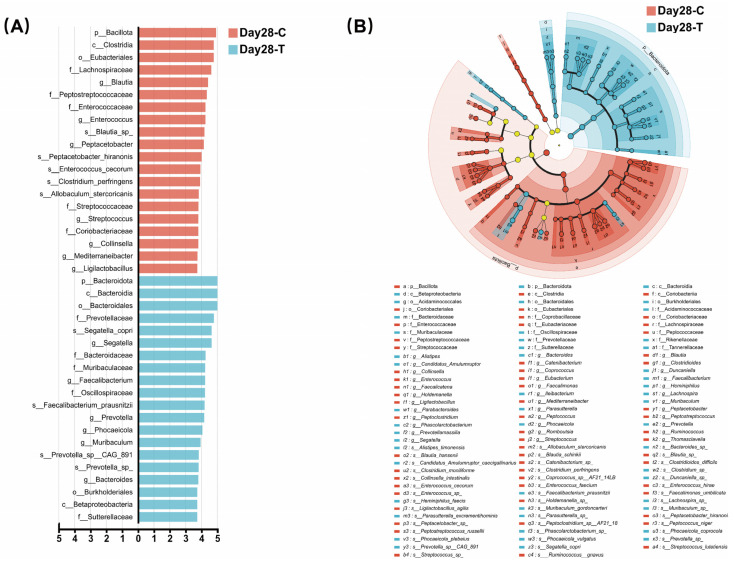
Linear discriminant analysis (LDA) effect size (LEfSe) of gut microbiota between the treatment (TRT, T) and control (CON, C) groups on day 28 (LDA score > 3). (**A**) Top 20 differential taxa between the two groups. (**B**) Evolutionary branching between the TRT group and the CON group. Circles radiating from the inside out in the evolutionary branching diagram represent taxonomic levels from phylum to species. Nodes represent taxa at each taxonomic level, and their size is proportional to relative abundance. Yellow nodes indicate species that do not differ significantly between the two groups.

To further identify microbial taxa that differed between the CON and TRT groups, LEfSe analysis was performed (Figure 6). LEfSe analysis (LDA score > 3) identified two differential phyla, three classes, three orders, ten families, thirteen genera, and nine species between the two groups.

In the CON group, Bacillota, Clostridia, Eubacteriales, five families (Coriobacteriaceae, Streptococcaceae, Enterococcaceae, Peptostreptococcaceae, and Lachnospiraceae), seven genera (*Ligilactobacillus*, *Mediterraneibacter*, *Collinsella*, *Streptococcus*, *Peptacetobacter*, *Enterococcus*, and *Blautia*), and five species (*Allobaculum stercoricanis*, *Clostridium perfringens*, *Enterococcus cecorum*, *Peptacetobacter hiranonis*, and *Blautia* sp.) were enriched.

In the TRT group, LEfSe analysis revealed a significant enrichment of the phylum Bacteroidota, along with families Oscillospiraceae and Prevotellaceae, which are widely recognized for their capacity to ferment complex dietary fibers. At the genus and species levels, the TRT group showed a higher abundance of known SCFA producers, most notably *Faecalibacterium prausnitzii*, *Prevotella* sp., and *Segatella copri*. Conversely, the CON group was enriched in Bacillota and specific pathobiont-associated taxa, such as *Clostridium perfringens* and *Enterococcus cecorum*.

#### 3.6.4. Functional Potential of the Gut Microbiota Based on KEGG Pathway Annotation

**Figure 7:**
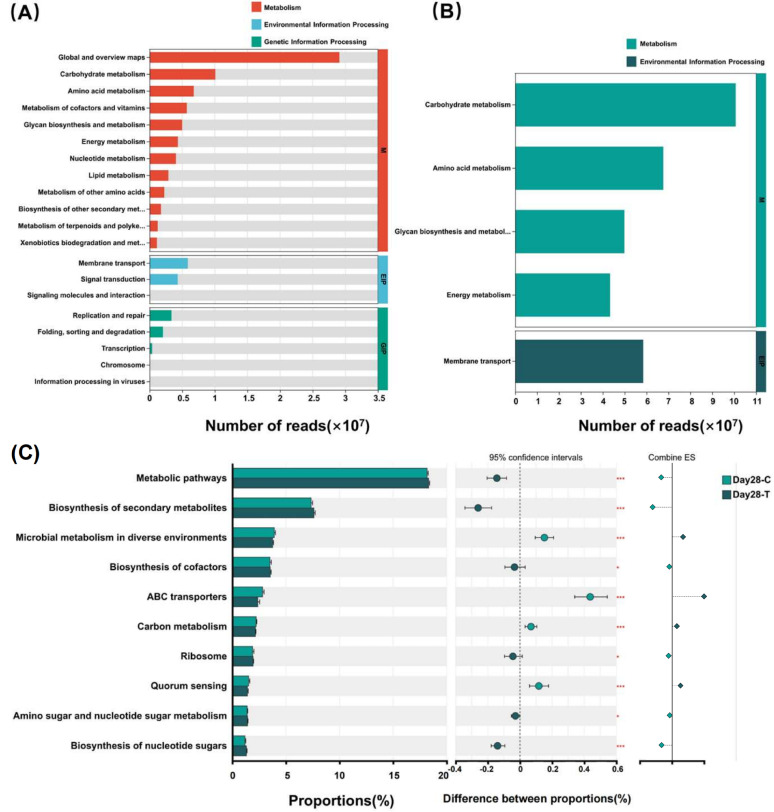
Functional pathways of the canine gut microbiota based on KEGG Level 1, Level 2, and Level 3 pathways between the treatment (TRT, T) and control (CON, C) groups on day 28. (**A**) KEGG Level 1 pathways. (**B**) KEGG Level 2 pathways. (**C**) KEGG Level 3 pathways. KEGG Level 1 and Level 2 pathways are displayed as the number of reads, whereas differential KEGG Level 3 pathways were identified using the Wilcoxon rank-sum test with false discovery rate (FDR) correction. In the Combine ES section of panel (C), diamonds indicate the direction of the effect size: light green diamonds represent pathways significantly enriched in the CON group, and dark green diamonds represent pathways significantly enriched in the TRT group. * *p* ≤ 0.05; *** *p* ≤ 0.001.

Functional annotation of the gut microbiota was performed using KEGG databases (Figure 7). At KEGG level 1, Metabolism was the most abundant category, followed by Environmental Information Processing and Genetic Information Processing (Figure 7A). Metabolism-related KEGG level 2 pathways included carbohydrate metabolism, energy metabolism, amino acid metabolism, glycan biosynthesis and metabolism, and metabolism of cofactors and vitamins. These pathways dominated the functional profile in both groups (Figure 7B). At KEGG level 3, the Wilcoxon rank-sum test with FDR correction was performed to compare functional pathways between the two groups on day 28. A total of 466 KEGG level 3 pathways were detected, of which 155 exhibited statistical significance (*p* < 0.05). Focusing on the primary functional shifts, the top 10 key pathways with the highest proportions are presented in Figure 7C, while the complete list of significantly different KEGG level 3 pathways is provided in Supplementary Table S3. Among the top 10 differential KEGG level 3 pathways, Metabolic pathways, Biosynthesis of secondary metabolites, Biosynthesis of cofactors, Ribosome, Amino sugar and nucleotide sugar metabolism, and Biosynthesis of nucleotide sugars were enriched in the TRT group, whereas Microbial metabolism in diverse environments, ABC transporters, Carbon metabolism and Quorum sensing were more abundant in the CON group.

#### 3.6.5. Carbohydrate-Active Enzyme Profiles Revealed by CAZy Annotation

**Figure 8:**
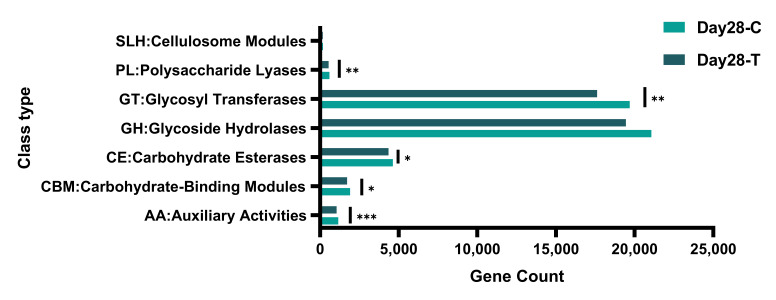
Distribution of carbohydrate-active enzyme (CAZy) classes based on gene counts in the canine gut microbiota between the treatment (TRT, T) and control (CON, C) groups on day 28. The relative abundances of seven CAZy classes-GHs, GTs, CEs, PLs, CBMs, AAs and SLHs-are shown based on gene counts across all samples. * *p* ≤ 0.05; ** *p* ≤ 0.01; *** *p* ≤ 0.001.

To further assess the carbohydrate utilization potential of the gut microbiota, CAZy annotation was performed. Based on annotation against the CAZy database, a total of 53,679 CAZyme-encoding genes were identified across all samples. These genes were mainly assigned to seven CAZy classes, including glycoside hydrolases (GHs), glycosyl transferases (GTs), carbohydrate-binding modules (CBMs), carbohydrate esterases (CEs), polysaccharide lyases (PLs), auxiliary activities (AAs), and cellulosome-related modules (SLHs). Among them, GHs and GTs accounted for the majority of CAZyme-encoding genes, whereas SLHs represented a minor fraction. Moreover, compared with the CON group, the TRT group exhibited significantly higher abundances in PLs, GTs, CEs, CBMs, and AAs (Figure 8).

Based on annotation against the CAZy database, a total of 34 CAZy families were identified as significantly different between the CON and TRT groups on day 28 (Table 4). These families were classified into 21 GHs, 4 GTs, 4 CEs, 4 CBMs, and 1 AA, highlighting the predominant involvement of carbohydrate-degrading and carbohydrate-modifying enzymes of intestinal microbial functions in response to the dietary intervention.

**Figure 9:**
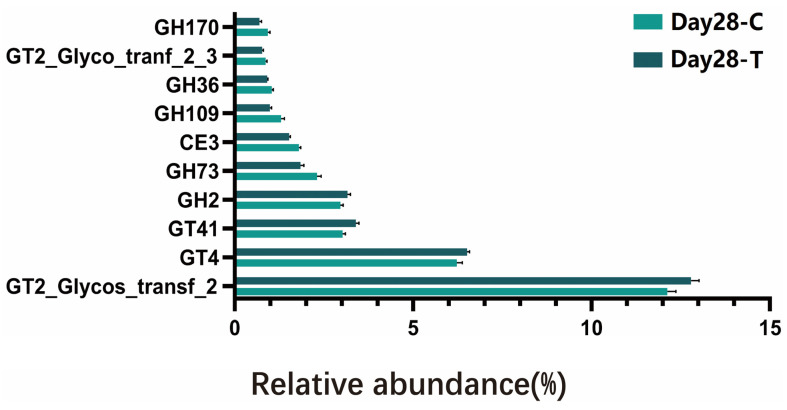
Relative abundances of the top 10 significantly different CAZy families between the control (CON, C) and treatment (TRT, T) groups on day 28. Differences were assessed using the Wilcoxon rank-sum test, and *p*-values were adjusted using the Benjamini-Hochberg false discovery rate (FDR) method. Values are expressed as mean ± SEM (CON, *n* = 18; TRT, *n* = 18).

To highlight the predominant functional changes, differential analysis focused on CAZy families with a mean relative abundance ≥0.1% in both groups on day 28 (Figure 9). The data revealed that the TRT group was significantly enriched in specific CAZy families functionally annotated for complex plant polysaccharide degradation. Notably, families responsible for the cleavage of complex glycosidic bonds as well as specific carbohydrate-binding modules exhibited significantly higher abundances in the TRT group compared to the CON group.

## 4. Discussion

Since their domestication, dogs have evolved from working animals into companion animals that contribute to their owners’ physical, psychological, and social well-being [13]. Accumulating evidence indicates that gut health in dogs can be modulated through dietary strategies, such as probiotics, prebiotics and microbial bioactive compounds [14,15]. Prebiotics and postbiotics have been recognized as effective approaches for improving intestinal health in human studies [16,17,18], and in vitro canine models provide supportive evidence [9]. According to the updated consensus definition, a prebiotic is “a substrate that is selectively utilized by host microorganisms conferring a health benefit” [19], whereas a postbiotic refers to “a preparation of inanimate microorganisms and/or their components that confers a health benefit on the host” [20].

In this study, the prebiotic and postbiotic blend was composed of baobab fruit pulp and acacia gum as prebiotics, and inactivated *Lactobacillus acidophilus* and distinct fractions of inactivated yeast strains as postbiotics. Baobab fruit pulp is characterized by a pleasant taste, high fiber content, and characteristic citrus aroma. Studies indicate that baobab fruit pulp is rich in antioxidants, minerals, and vitamins, conferring multiple nutritional and medicinal properties, and it serves as a traditional food and source of income in parts of Africa [21]. Compared with other prebiotics, baobab fruit pulp powder contains polysaccharides with a unique composition that can significantly increase the levels of short-chain fatty acids in the gut [22]. Acacia gum, also named arabic gum, is a high-molecular-weight glycoprotein and polysaccharide complex extracted from Acacia species and is regarded as a natural prebiotic with positive effects in a variety of experimental models, such as humans, chickens, and mice [8,23]. Yeast cell walls are rich in β-glucans, which play important roles in maintaining gut barrier function and regulating microbial balance [24]. Additionally, yeast-derived single-cell proteins provide essential amino acids and can indirectly promote gut health by improving the intestinal environment [25,26]. Owing to its exceptional resistance to acid and bile salts, *Lactobacillus acidophilus* is widely recognized and frequently recommended as a highly effective dietary probiotic. [27]. As a postbiotic, it has demonstrated significant gut barrier protection and immunomodulatory effects in broiler chickens [10]. Recent review by Niranjana and colleagues has shown that *Lactobacillus acidophilus* plays a pivotal role in maintaining canine gut health by promoting microbiota balance and enhancing gastrointestinal function. [28].

It is crucial to acknowledge a specific feature of our study design regarding the experimental timeline. The administration of the prebiotic and postbiotic supplement commenced at the beginning of the 7-day dietary transition (pre-trial period), whereas the “day 0” sampling was conducted at the end of this 7-day phase. Consequently, the day 0 values do not represent a strictly untreated physiological baseline, but rather the state after one week of dietary adaptation and concurrent supplementation. This design choice was intentionally made to evaluate the supplement’s efficacy in mitigating the acute gastrointestinal stress associated with an abrupt diet change. Indeed, significant differences in fecal scores were observed between the two groups on days 3 and 4 during the 7-day dietary transition period. This may indicate a potential relationship between the recovery of fecal consistency and the administration of the supplement. However, because the intervention coincided with the period during which all dogs adapted to the new diet, these transient differences might reflect a complex interaction between the new diet and the supplement, rather than a singular effect. This also presents potential avenues for future research.

Similarly, at the start of the formal experiment (day 0), there were no significant differences between groups in indicators of intestinal inflammation or barrier function. However, on day 28, the TRT group exhibited significantly lower fecal calprotectin level, a marker of intestinal inflammation [29], and serum DAO concentration, a marker of intestinal barrier permeability [30]. It is important to note that because the dogs enrolled in this study were clinically healthy, the absolute values of these biomarkers likely remained within normal physiological ranges throughout the trial. Therefore, while these markers are informative, their reduction alone does not prove definitive barrier restoration or inflammation resolution. Rather, the magnitude of these significant reductions supports a possible beneficial effect of the supplement on these selected biomarkers of intestinal barrier function and local inflammation. Fecal odor can serve as an indirect indicator of canine gut health when dogs consume indigestible diets, experience gastrointestinal dysfunction, or suffer from microbial dysbiosis [31]. The odor of feces is closely associated with microbial metabolites, such as indole and phenolic compounds, which are products derived from bacterial amino acid metabolism [32]. In contrast to the statistically significant alterations observed in calprotectin and DAO, fecal indole levels in the TRT group only exhibited a statistical trend towards reduction compared with the CON group at the end of the study. This indicates a subtle, though not definitively established, reduction in microbial tryptophan metabolism associated with the supplement.

SCFAs, produced by gut microbiota through the fermentation of carbohydrates, are essential for maintaining intestinal health [33]. Acetate, propionate, and butyrate are the most abundant SCFAs in the gut and collectively account for more than 90% of total SCFA production [34]. Among them, butyrate plays a crucial role in maintaining epithelial barrier integrity and attenuating intestinal inflammation [35,36,37,38]. Therefore, SCFA concentrations can serve as indicators of microbial balance and intestinal homeostasis. In the present study, fecal butyrate levels were significantly higher in the TRT group than in the CON group, suggesting that the supplement enhanced colonic butyrate production or accumulation. The biological relevance of this finding is robustly supported by previous canine-specific literature. For example, as demonstrated by Sandri et al. [5], dietary interventions utilizing specific fermentable fiber sources can significantly modulate gut microbiota activity and enhance SCFA production in dogs. Our results align with this paradigm, further contextualizing how the fermentable prebiotics in our supplement provide essential substrates for microbial fermentation. The resultant increase in butyrate production is associated with, and plausibly contributes to, the observed improvements in intestinal barrier markers and the attenuation of local inflammation. While our data provide a biologically coherent associative link supported by existing literature, further functional studies are required to definitively establish a causal mechanism. Nevertheless, these findings highlight a potential active metabolic modulation achieved by this nutritional intervention.

Members of the phylum Bacteroidota play important roles in intestinal metabolism, including nitrogen utilization, carbohydrate fermentation, and the transformation of bile acids and other steroids [39]. Previous studies have shown that dogs with inflammatory bowel disease have a reduced abundance of Bacteroidota compared with healthy ones [1]. In the present study, the higher abundance of Bacteroidota observed on day 28 in the TRT group is highly consistent with an increased utilization of fermentable substrates introduced by the supplement. At the family level, Oscillospiraceae, Prevotellaceae, and Akkermansiaceae were more abundant in the TRT group than in the CON group on day 28. Oscillospiraceae abundance has been negatively associated with systemic inflammation [40]. Prevotellaceae, which predominantly colonize the colon, contribute to intestinal barrier integrity through SCFA production [41,42]. *Akkermansia muciniphila* enhances mucosal barrier function by maintaining mucus layer structure and tight-junction integrity, thereby supporting epithelial health and reducing intestinal inflammation [43]. Dietary supplementation was associated with significant alterations in the composition of the gut microbiota in the TRT group. These microbial shifts parallel the observed improvements in indices of intestinal barrier and inflammation, suggesting a potential associative link rather than direct mechanistic proof. Furthermore, it is highly noteworthy that despite these robust microbial and metabolite changes, macroscopic clinical parameters such as fecal scores remained stable during the main trial period. Although a statistical difference in body weight was observed on day 28 after adjusting for baseline variance, the body weights of all subjects fluctuated strictly within a healthy physiological range, with no pathological weight loss recorded. This lack of an adverse macroscopic clinical response is anticipated, given that the enrolled subjects were already clinically healthy with optimal gastrointestinal function at baseline. Biologically, the stability of these clinical indices confirms that the supplement is well-tolerated and does not disrupt normal physiological processes. Clinically, it implies that the benefits of this supplementation in healthy animals are preventive rather than therapeutic. Therefore, these microbiota shifts reflect a biological optimization of the gut microenvironment rather than a visible therapeutic resolution. Importantly, because the body weight and overall dietary intake of the dogs remained completely stable and consistent throughout the study, and the minor weight variation lacked clinical significance, these marked microbiota and metabolic shifts can be interpreted as primarily driven by the targeted efficacy of the prebiotic–postbiotic supplementation rather than general nutritional differences.

CAZymes encompass several classes of enzymes involved in the assembly, degradation, and modification of oligo- and polysaccharides [44]. In the present study, the dietary supplementation did not induce a uniform increase or decrease in overall CAZy abundance, but instead resulted in a redistribution of CAZy family composition. Although GHs were enriched in both groups, the specific GH families showing enrichment differed markedly between the CON and the TRT groups. Notably, 15 CAZy families were enriched in the TRT group, whereas 19 families were enriched in the CON group, indicating that the two dietary regimens promoted distinct carbohydrate utilization profiles rather than a shared metabolic response.

Specifically, GH13 subfamilies (e.g., GH13_4, GH13_9, GH13_14, and GH13_39) were enriched in the TRT group, suggesting an enhanced capacity for α-glucan and starch degradation [45,46,47,48,49], which facilitates SCFA fermentation. Concurrently, the significant enrichment of other diverse glycoside hydrolases (e.g., GH14, GH51, GH95), along with specific carbohydrate esterases and carbohydrate-binding modules (CBM9, CBM13), indicates a highly coordinated microbial enzymatic system [50,51,52,53,54]. Rather than acting in isolation, these accessory modules and esterases enhance enzyme–substrate interactions and dismantle complex structural linkages [55,56], thereby facilitating the degradation of complex dietary fibers such as pectin and arabinogalactans. Together with auxiliary activities involved in plant cell wall biotransformation (e.g., AA7) [57], the cooperative enrichment of these distinct CAZy classes provides a robust mechanistic basis for the enhanced complex carbohydrate utilization and subsequent butyrate production observed in the TRT group.

Compared with the TRT group, the CAZy pathways in the CON group exhibited a distinct enrichment pattern. In the CON group, a distinct set of CAZy families was significantly enriched, mainly comprising glycoside hydrolases involved in the utilization of relatively simple or readily accessible carbohydrates, together with a limited number of carbohydrate esterases, carbohydrate-binding modules, and one auxiliary activity enzyme. Notably, several members of the GH13 subfamily (including GH13_18, GH13_39, GH13_11, GH13_29, and GH13_4) were enriched in the CON group. Similar to the TRT group, these GH13 subfamilies are closely associated with the degradation of α-glucans and starch-related substrates. The presence of these shared GH13 pathways suggests that the gut microbiome tends to preserve core metabolic functions related to the utilization of dietary starches and endogenous glycans in the absence of additional fermentable fiber supplementation.

In addition to GH13 subfamilies, the CON group showed enrichment of GH2, GH36, GH42, and GH65, which are commonly implicated in the hydrolysis of oligosaccharides and disaccharides, such as lactose- and galactose-containing carbohydrates [58,59]. Together with GH4 and GH170, these enzymes are often considered a part of a more conserved or “baseline” carbohydrate utilization repertoire, supporting routine saccharide turnover rather than the extensive breakdown of structurally complex polysaccharides [60]. This functional profile is consistent with a gut microbial ecosystem relying primarily on conventional carbohydrate sources provided by the basal diet and the placebo chew.

Interestingly, the enrichment of CE3 and CE19 in the CON group further suggests a modest capacity for de-esterification reactions, although these activities appeared less coordinated with polysaccharide-degrading hydrolases compared with the TRT group. Moreover, the presence of CBM32 among CON-enriched families indicates that carbohydrate-binding capacity was not absent but rather skewed toward CBM types associated with different substrate specificities. This observation implies that the functional divergence between groups was not driven by the overall abundance of CBMs per se, but by a shift in CBM composition and their associated catalytic partners.

In addition to GHs and CEs, several GT families were differentially enriched between the groups, including GT2, GT4, and GT41. GTs catalyze the transfer of sugar moieties from activated donor molecules to acceptor substrates, forming glycosidic bonds [61]. Unlike GHs and CEs, which primarily mediate the hydrolysis of dietary polysaccharides, GTs are mainly involved in the biosynthesis and modification of cell-surface glycans, extracellular polysaccharides, and structural carbohydrates. Within the CAZy database, GT2 and GT4 represent widely distributed and functionally important GT families, participating in the assembly and modification of diverse bacterial polysaccharides [62]. Their differential enrichment between groups suggests that, alongside altered polysaccharide degradation pathways, the gut microbiota may undergo adaptive modifications of cell-surface and extracellular polysaccharide structures in response to dietary supplementation. Therefore, changes in GT abundance are more likely to reflect microbial physiological adaptation and cell envelope remodeling in response to altered nutrient availability, rather than direct polysaccharide breakdown.

The enrichment of specific GT families in the TRT group may indicate enhanced synthesis of cell-associated or extracellular glycans, potentially facilitating microbial colonization, biofilm formation, or interactions with complex dietary fibers. However, given their indirect role in carbohydrate catabolism, GT-related pathways were not considered primary contributors to the observed enhancement of fiber utilization capacity in this study. In contrast, CAZy families enriched in the CON group included multiple glycoside hydrolases and the auxiliary activity family AA7, which may reflect a preferential reliance on endogenous substrates or simpler carbohydrate sources in the absence of supplemental fiber. This bidirectional pattern of CAZy enrichment highlights distinct carbohydrate utilization strategies adopted by the gut microbiota under different dietary conditions, emphasizing functional differentiation rather than global enhancement of carbohydrate metabolism.

Overall, the findings of the present study indicate that supplementation with the prebiotic–postbiotic blend was associated with a pronounced reshaping of the gut microbial community structure in the TRT group, including increased relative abundances of taxa involved in carbohydrate fermentation and mucosal homeostasis. These findings suggest the presence of two coherent diet-microbiota-metabolite-host axes. Specifically, the supplement selectively modulated the gut microbiota of the treated dogs, leading to an enrichment of KEGG pathways and CAZy enzyme repertoires associated with the degradation of pectin- and arabinogalactan-rich substrates. These effects were accompanied by a significant increase in fecal butyrate concentrations. In parallel, under conditions of enhanced complex carbohydrate availability, tryptophan metabolism–related pathways in the TRT group exhibited a trend toward reduction, coinciding with a subtle decrease in fecal indole levels. Taken together, these results suggest that dietary interventions using complex, fermentable carbohydrates such as those found in baobab fruit pulp and acacia gum, and the selected postbiotics present in the supplement, can selectively modulate gut microbial composition and functional capacity. These microbial shifts coincide with the production of beneficial metabolites, which may in turn support intestinal barrier integrity and immune homeostasis. Importantly, because the dogs enrolled in this study were already healthy, these robust microbiota shifts toward carbohydrate fermentation and the concurrent modulation of inflammatory markers should not be interpreted as a therapeutic improvement in health status. Rather, they represent a favorable metabolic shift and an optimization of the gut microenvironment that proactively supports and maintains intestinal homeostasis.

From a broader perspective, it is highly relevant to contextualize these findings within a One Health and environmental microbiology framework. The canine gut ecosystem does not exist in isolation; rather, it is continuously shaped by environmental and management-related microbial exposures. As demonstrated by Raspa et al. [3], factors such as diet type and feeding hygiene can significantly influence microbial contamination and potentially interact with the host gut microbiota. In this context, optimizing internal gut health through targeted nutritional interventions may serve as an essential defense mechanism to support intestinal resilience against continuous external microbial challenges encountered in the domestic environment.

Finally, several limitations of the present study should be explicitly acknowledged. First, the “day 0” baseline sampling occurred after a 7-day simultaneous dietary adaptation and intervention period. Consequently, these values reflect an already adapting state rather than a strict, unexposed physiological baseline. Second, the duration of the formal experimental period was relatively short (28 days), which limits our ability to evaluate the long-term stability of the observed microbial and metabolic shifts. Third, while body weights remained stable throughout the study, precise quantitative daily feed intake data were not individually recorded, precluding deeper analysis of diet-microbiome interactions. Fourth, our physiological assessments relied on indirect biomarker-based inferences alongside limited clinical endpoints (fecal scores). We did not perform direct evaluations of intestinal morphology or functional permeability testing, which are necessary to definitively confirm mucosal restoration. Fifth, the functional profiles derived from metagenomic sequencing reflect the genetic potential and metabolic capacity of the microbiome, rather than its actual in vivo activity or gene expression [4]. Finally, the clinical relevance of our findings is inherently limited by the cohort. Because the dogs were clinically healthy rather than suffering from gastrointestinal disease, and their fecal scores were already optimal by day 0, our results demonstrate a biological optimization. This cannot be directly extrapolated to represent a meaningful therapeutic benefit for dogs with active chronic enteropathies or unstable stools. Future large-scale, long-term studies incorporating direct intestinal evaluations and clinical disease cohorts are warranted to fully elucidate the clinical efficacy of this prebiotic–postbiotic supplementation.

## 5. Conclusions

Supplementation with the prebiotic and postbiotic during the dietary transition was associated with a temporary improvement in stool consistency. However, this should be interpreted with caution due to the concurrent diet change. It also significantly reduced fecal calprotectin and serum diamine oxidase levels, increased fecal butyrate concentration and elevated the relative abundance of beneficial microbial taxa, including Bacteroidota, Oscillospiraceae, Prevotellaceae and *Prevotella*. Crucially, CAZy pathway analysis revealed a specific enhancement in microbial enzymatic capacities for degrading complex plant polysaccharides, particularly pectin and starch, which provides a plausible mechanistic basis for the observed increase in butyrate production. Collectively, the data support a promising short-term effect on selected biomarkers and microbiota structure and function in healthy dogs under the described conditions. The tested supplement showed potential to modulate gut-associated markers and microbial functions; however, further studies in dogs with gastrointestinal disturbances and longer follow-up periods are warranted.

## Figures and Tables

**Table 1:** Ingredient composition and nutrient levels of the basal diet.

Ingredient Composition	%	Nutrient Content ^2^	
Poultry meal	39.23	Gross energy, kcal/g	4.73
Soy protein concentrate	20.00	Dry matter, %	91.27
Barley	10.00	Crude protein, %	40.37
Beet pulp	10.00	Acid-hydrolyzed fat, %	14.11
Rice	7.00	Crude fiber, %	4.91
Chicken fat	5.00	Ash, %	7.98
Tapioca	4.00		
Powder palatant	3.00		
Salt	0.50		
Premix ^1^	1.27		
Total	100.00		

^1^ Premix provided the following per kilogram of feed: vitamin A, 15,000 IU; vitamin B_1_, 30 mg; vitamin B_2_, 28 mg; vitamin B_3_ (niacin), 110 mg; vitamin B_5_ (pantothenic acid), 85 mg; vitamin B_6_, 12 mg; vitamin B_12_, 0.19 mg; vitamin D_3_, 1500 IU; vitamin E, 300 IU; copper (CuSO_4_), 8.3 mg; iron (FeSO_4_), 80 mg; iodine (CaI_2_), 1.5 mg; manganese (MnSO_4_), 18 mg; zinc (ZnSO_4_), 120 mg; selenium (Na_2_SeO_4_), 0.26 mg. ^2^ The nutrient levels of the diets were analyzed.

**Table 2:** Fecal scoring results during the pre-trial period.

Items	Control Group	Experimental Group	*p*-Value
Pre-trial Day 1	3.39 ± 0.10	3.42 ± 0.10	0.87
Pre-trial Day 2	3.28 ± 0.12	2.97 ± 0.14	0.12
Pre-trial Day 3	3.31 ± 0.13	2.83 ± 0.13	0.03
Pre-trial Day 4	3.22 ± 0.13	2.78 ± 0.12	0.04
Pre-trial Day 5	3.00 ± 0.13	2.78 ± 0.12	0.22
Pre-trial Day 6	2.92 ± 0.14	2.86 ± 0.12	0.68
Pre-trial Day 7	2.78 ± 0.14	2.75 ± 0.16	0.88

Note: CON: the control group (*n* = 18); TRT: the treatment group (*n* = 18).

**Table 3:** Fecal Score and body weight in dogs.

Group	Day 0		Day 28	
	Fecal Score	Body Weight	Fecal Score	Body Weight
CON	2.78 ± 0.14	14.70 ± 1.22	2.53 ± 0.13	15.49 ± 1.25 ^a^
TRT	2.75 ± 0.16	15.10 ± 1.33	2.47 ± 0.12	15.26 ± 1.37 ^b^
*p*-value	0.90	0.80	0.75	<0.001

Note: CON: the control group (*n* = 18); TRT: the treatment group (*n* = 18). ^a,b^ Different superscript letters denote a statistically significant difference (*p* < 0.05).

**Table 4:** Differentially abundant CAZy families between the CON group and TRT groups on day 28.

Name	CON	TRT	*p*-Value
GT2_Glycos_transf_2	12.12 ± 0.241	12.80 ± 0.218	0.05
GT4	6.224 ± 0.152	6.511 ± 0.072	0.03
GT41	3.029 ± 0.076	3.396 ± 0.087	<0.01
GH2	2.964 ± 0.077	3.158 ± 0.081	0.04
GH73	2.308 ± 0.113	1.848 ± 0.095	<0.01
CE3	1.799 ± 0.054	1.528 ± 0.034	<0.01
GH109	1.301 ± 0.096	0.986 ± 0.045	0.01
GH36	1.038 ± 0.044	0.913 ± 0.017	<0.01
GT2_Glyco_tranf_2_3	0.865 ± 0.041	0.769 ± 0.032	0.04
GH170	0.928 ± 0.057	0.698 ± 0.052	0.01
CE19	0.449 ± 0.030	0.549 ± 0.017	0.04
GH4	0.537 ± 0.031	0.343 ± 0.024	<0.01
GH13_18	0.461 ± 0.040	0.317 ± 0.025	<0.01
GH13_39	0.410 ± 0.021	0.283 ± 0.018	<0.01
GH51_1	0.392 ± 0.032	0.279 ± 0.012	<0.01
CE11	0.297 ± 0.049	0.352 ± 0.023	0.04
GH24	0.245 ± 0.025	0.376 ± 0.037	0.01
GH42	0.341 ± 0.022	0.276 ± 0.015	0.01
GH65	0.301 ± 0.028	0.220 ± 0.018	0.01
GH13_14	0.225 ± 0.013	0.289 ± 0.007	<0.01
GH16_3	0.196 ± 0.033	0.293 ± 0.021	<0.01
GH13_11	0.281 ± 0.021	0.171 ± 0.013	<0.01
CE14	0.264 ± 0.029	0.152 ± 0.011	<0.01
GH146	0.180 ± 0.012	0.224 ± 0.008	<0.01
GH13_29	0.227 ± 0.025	0.165 ± 0.012	0.04
GH43_12	0.164 ± 0.012	0.208 ± 0.011	0.01
CBM13	0.135 ± 0.015	0.208 ± 0.015	<0.01
AA7	0.214 ± 0.025	0.126 ± 0.022	0.01
CBM32	0.196 ± 0.021	0.127 ± 0.010	<0.01
GH106	0.135 ± 0.007	0.184 ± 0.009	<0.01
GH13_4	0.184 ± 0.012	0.120 ± 0.013	<0.01
CBM48	0.184 ± 0.030	0.114 ± 0.011	<0.01
GH140	0.122 ± 0.009	0.161 ± 0.012	0.02
CBM9	0.116 ± 0.012	0.155 ± 0.011	0.04

Note: Differential CAZy families were identified using the Wilcoxon rank-sum test with false discovery rate (FDR) correction between the treatment (TRT) and control (CON) groups on day 28. Only CAZy families with a mean relative abundance ≥0.1% in both groups on day 28 were included. Data are presented as mean ± SEM (CON, *n* = 18; TRT, *n* = 18).

## Data Availability

The original contributions presented in this study are included in the article/Supplementary Materials. Further inquiries can be directed to the corresponding author.

## Supplementary Materials

The following supporting information can be downloaded at: https://www.mdpi.com/article/10.3390/vetsci13050417/s1, Table S1: Detailed baseline characteristics of the dogs in the treatment and control groups; Table S2: Significantly differential CAZy pathways between the treatment and control groups on day 28; Table S3: Significantly differential KEGG Level 3 pathways between the treatment and control groups on day 28.
